# A CCAAT-binding factor, SlNFYA10, negatively regulates ascorbate accumulation by modulating the d-mannose/l-galactose pathway in tomato

**DOI:** 10.1038/s41438-020-00418-6

**Published:** 2020-12-01

**Authors:** Weifang Chen, Tixu Hu, Jie Ye, Bing Wang, Genzhong Liu, Ying Wang, Lei Yuan, Jiaming Li, Fangman Li, Zhibiao Ye, Yuyang Zhang

**Affiliations:** 1 Key Laboratory of Horticultural Plant Biology, Ministry of Education, Huazhong Agricultural University, 430070 Wuhan, China; 2 HZAU Chuwei Institute of Advanced Seeds, 430070 Wuhan, China

**Keywords:** Plant molecular biology, Metabolomics

## Abstract

Ascorbic acid (AsA), an important antioxidant and growth regulator, and it is essential for plant development and human health. Specifically, humans have to acquire AsA from dietary sources due to their inability to synthesize it. The AsA biosynthesis pathway in plants has been elucidated, but its regulatory mechanism remains largely unknown. In this report, we biochemically identified a CCAAT-box transcription factor (SlNFYA10) that can bind to the promoter of *SlGME1*, which encodes GDP-Man-3’,5’-epimerase, a pivotal enzyme in the d-mannose/l-galactose pathway. Importantly, SlNFYA10 simultaneously binds to the promoter of *SlGGP1*, a downstream gene of *SlGME1* in the d-mannose/l-galactose pathway. Binding assays in yeast and functional analyses in plants have confirmed that SlNFYA10 exerts a negative effect on the expression of both *SlGME1* and *SlGGP1*. Transgenic tomato lines overexpressing *SlNFYA10* show decreased levels of *SlGME1* and *SlGGP1* abundance and AsA concentration in their leaves and fruits, accompanied by enhanced sensitivity to oxidative stress. Overall, *SlNFYA10* is the first CCAAT-binding factor identified to date to negatively regulate the AsA biosynthetic pathway at multiple sites and modulate plant responses to oxidative stress.

## Introduction

Ascorbic acid (AsA, also referred to as vitamin C) contributes to nutritional quality and stress responses in plants and thus has attracted increased amounts of attention. Since humans and higher primates have lost the capability to synthesize AsA, this compound must be obtained from dietary sources rich in AsA, e.g., fresh vegetables and fruits^1^. AsA plays an important role in protecting human health and in disease prevention. For example, AsA can effectively control tumor progression^2^, and intracellular AsA helps maintain the integrity and function of several processes in the central nervous system^3^. In plants, AsA can remove reactive oxygen species produced by photosynthesis, respiration, biological stress, and abiotic stress so that plants can carry out normal physiological processes. For AsA biosynthesis, the major biosynthesis pathway is the d-mannose/l-galactose pathway (Wheeler & Smirnoff pathway), although an alternative pathway in plants involving galacturonate has been proposed^4,5^. However, controversy exists between the d-glucose^6^ and myo-inositol pathways^7^. Between them, the pivotal biosynthetic pathway of d-mannose/l-galactose involves ten catalytic steps through which d-glucose is converted to AsA. The first six steps involved the synthesis of active riboside sugars, which are utilized as substrates for AsA synthesis, as well as precursors of cell wall polysaccharides and glycoproteins. The last four steps, starting from GDP-l-galactose, constitute the exclusive steps for AsA synthesis. The genes in the d-Man/l-Gal pathway have been identified; they encode GDP-d-Man pyrophosphorylase (GMP)^8^, GDP-Man-3’,5’-epimerase (GME)^9^, GDP-l-Gal phosphorylase (GGP)^10^, l-Gal-1-P phosphatase (GPP)^11^, l-Gal dehydrogenase (GalDH)^12^, and l-galactono-1,4-lactone dehydrogenase (GLDH)^13^. GME converts GDP-d-mannose into GDP-l-galactose, yielding an alternative product of GDP-l-glucose. In most plants, there is a single copy of the *GME* gene^14^, whereas in tomato (*Solanum lycopersicum*), two *GME* genes named *GME1* and *GME2* have been found^15^. Overexpression of *GME1* and *GME2* could increase the AsA content, while knockdown of either *GME* gene decreased the AsA content^16^. Simultaneous suppression of the *GME1* and *GME2* genes by RNA interference (RNAi) could reduce the AsA content by 40–60%, with increasing cell wall mannose content and fragility^17^. Suppressed expression of *GME1* or *GME2* separately revealed different functions in cell wall synthesis^18^, suggesting the divergence of functions of *GME* family members.

In plants, the AsA content is affected by various factors, such as light^19,20^, temperature^21^, minerals^22^, and environmental cues. AsA accumulation is also regulated by hormones such as auxin^23^, methyl jasmonate^24^, jasmonic acid^25^, and Abscisic Acid (ABA)^26^. Transcription factors or regulators purportedly modulate AsA biosynthesis. *amr1* (Ascorbic acid Mannose Pathway Regulator 1), an activation-tagged Arabidopsis (*Arabidopsis thaliana*) ozone-sensitive mutant, showed decreased AsA content when the expression of genes in the d-Man/l-Gal pathway were negatively regulated^27^. The photomorphogenic factor COP9 signalosome subunit 5B (CSN5B) could decrease the AsA content by modulating the protein abundance of the AsA biosynthetic enzyme GDP-Man-pyrophosphorylase (VTC1)^28^. Arabidopsis AtERF98 and the tomato HD-Zip I family transcription factor (SlHZ24) could bind to the *VTC1* promoter and increased the AsA content by positively regulating *VTC1* expression^29,30^. Two nucleotide sugar pyrophosphorylase-like proteins from Arabidopsis, KONJAC1 (KJC1) and KJC2, could stimulate GMP enzymatic activity and increase AsA accumulation^31^. Moreover, a Cys2/His2-type zinc-finger protein named SlZF3 could directly interact with CSN5B, preventing CSN5B binding to GMP and thus increasing the AsA content in tomato^32^. More recently, an upstream open reading frame (uORF) in the long untranslated region of *GGP* was demonstrated to encode a peptide that functions in the feedback regulation of AsA at the posttranscriptional level^33^. To date, the regulation of AsA biosynthesis largely occurs at the VTC1-catalyzed step^28–30,32^, while the regulation of other genes in the AsA synthesis pathway deserves further investigation.

NFYAs belong to the NFY (Nuclear Factor Y, also known as Heme activator protein or CCAAT-binding factor) complex, which contains NFYAs, NFYBs and NFYCs, and function as heterodimers. After dimerization between NFYB and NFYC in the cytoplasm, NFYA is further recruited to form a heterotrimer. NFYA binds to the CCAAT-box to regulate the expression of downstream genes^34^. Sequences associated with the NFY heterotrimer complex have been found in all the sequenced genomes of eukaryotes^35^, and there are one or two genes encoding NFYs in mammals and yeast^36^. However, in plants, the NFY family has undergone expansion^34^. The NFY complex was reported to be involved in the regulation of plant growth and development and the stress response. NFYA in wheat positively responds to low nitrogen and phosphorus: overexpression of *TaNFYA-B1* stimulated root development by upregulating nitrate and phosphate transporters and, subsequently, nitrogen and phosphorus absorption in roots^37^. Overexpression of the transcription factor *NFYC9* mediates ABA signaling by targeting ABA-responsive transcription factors such as ABI5^38^. The expression of *NFYA5* was strongly induced by drought in an ABA-dependent manner, and *NFYA5* overexpression could reduce moisture loss and drought sensitivity^39^. However, overexpression of the wheat *NFYA10* gene increased plant sensitivity to salinity, as judged on the basis of seed germination and root growth^40^.

NFY factors have been known to function as heterotrimer complexes during various processes of plant growth and development^41–43^, although only a single NFY transcription factor has been reported to be involved in the stress response in plants. However, the regulatory mechanism through which metabolites, e.g., AsA, accumulate has not been elucidated. In this study, an NFYA transcription factor was determined to negatively regulate AsA biosynthesis at multiple sites in the d-mannose/l-galactose pathway.

## Materials and methods

### Yeast one-hybrid

A yeast one-hybrid assay was carried out via a Matchmaker One-Hybrid Library Construction and Screening Kit (Clontech, http://www.clontech.com/) according to the manufacturer’s instructions. A 2-kb promoter fragment of *SlGME1* from the initiation site, as well as a mutated fragment of the *SlGME1* promoter were amplified from tomato genomic DNA and then cloned into a pAbAi vector (Clontech). The full-length and truncated NFYA CDSs were amplified from tomato complementary DNA (cDNA) via PCR and cloned into a pGADT7 vector (Clontech); the primer sequences used are listed in Supplementary Table S2. Both the pHIS2.0 bait vector and the pGADT7 prey vector were introduced into Y1H Gold yeast (Clontech) and cultured on SD/-Leu-Ura media. After 3 days, the positive yeast strains were selected and diluted in sterilized distilled water to an OD_600_ of 0.1, and 2.5 μl of suspension was spotted onto SD/-Leu-Ura media, with or without AbA (Clontech). The plates were subsequently incubated for 3–7 days at 30 °C.

### Transient expression in tobacco leaves by agro infiltration

There are four CCAAT-boxes in the 2-kb promoter of *SlGME1*, and different 5’-deleted *SlGME1* promoter fragments were amplified with specific primers (Supplementary Table S2). These fragments were cloned into a pGreenII 0800-LUC reporter vector. The full-length ORF of NFYA was amplified from tomato cDNA and inserted into a pGreenII 62-SK effector vector, which was subsequently introduced into *A. tumefaciens* strain GV3101 cells together with a pSoup helper vector. The *Agrobacterium* cells were subsequently activated by treatment with AI buffer (10 mmol/l MES, 10 mmol/l MgCl_2_, 150 μmol/l AS). The *Agrobacterium* cells with different vectors were mixed together and injected into young tobacco (*Nicotiana benthamiana*) leaves for transient expression, after which they were were evaluated after a 2-day incubation period^44^. Luciferase reporter genes were assayed using a Dual-Luciferase Reporter Assay System (Promega, http://www.promega.com/) using an Infinite M200 Pro instrument (Tecan).

A 35S::UAS-GUS reporter system was used to characterize the transcriptional activity of SlNFYA10, as described by Wang et al.^45^. The CDS of SlNFYA10 was amplified and linked to a pYF503 vector, generating a GDBD-SlNFYA10 effector construct. The effector construct, empty control (pYF503, designated GDBD), and 35S::UAS-GUS reporter plasmid were then transformed into *A. tumefaciens* strain GV2260. The reporter and two effectors were mixed together, after which the mixture injected into tobacco leaves, followed by GUS staining after a 48 h incubation period. GUS staining was performed as described previously^46^.

### Vector construction and tomato transformation

Full-length NFYA ORFs and RNAi fragments were amplified via PCR using Phanta polymerase (Vazyme, Nanjing, China) from tomato cDNA using gene-specific primers (Supplementary Table S2). The full-length SlNFYA10 ORF and RNAi fragment were then cloned into a pMV3 overexpression vector (modified from pHELLSGATE8) and pHELLSGATE8 (Invitrogen), respectively, via recombination using Exnase II. These vectors were transferred into *A. tumefaciens* strain C58 for tomato transformation. The constructs were subsequently transformed into Ailsa Craig (AC) tomato, and transgenic plants were confirmed via PCR using genomic DNA from the leaves; the primers used are shown in Supplementary Table S2.

### Gene expression analysis

Total RNA was extracted from roots, stems, leaves, and flowers as well as immature fruit, green fruit, breaker-stage fruit, and red ripe fruit using TRIzol reagent. The RNA was then reverse transcribed into cDNA using a HiScript II 1st Strand cDNA Synthesis Kit (Vazyme), and the gene expression in these tissues was measured. Quantitative PCR (qPCR) was performed using a SYBR Light Cycler 480 instrument in conjunction with SYBR Green I Master Kit (Roche, http://www.roche.com/) according to the manufacturer’s protocols. The RNA of the transgenic plants and wild type was extracted and reverse transcribed to cDNA to determine the expression of *SlNFYA10*, *SlGME1*, *SlGME2*, and other genes in the AsA biosynthesis pathway. The sequences of the primers used are listed in Supplementary Table S3. Three biological replicates for the transgenic lines and the wild type were evaluated. The relative expression of specific genes was quantified using the 2^−ΔΔ^^Ct^ method, and the *actin* gene was used as a constitutive internal control.

### Ascorbic acid assays

The AsA levels were measured in T_1_ transgenic lines, as well as wild-type plants as previously described^47^. Leaf samples were taken from the third or fourth leaf from the top of 1-month-old tomato plants. The fruit samples were taken at the red ripe stage. The samples were collected and ground to a fine powder in liquid nitrogen. Approximately 0.2 g of frozen leaf tissue and 0.3 g of frozen fruit tissue were added to one milliliter of ice-cold 6% trichloroacetic acid in a 2-ml Eppendorf tube. Each homogenate was centrifuged at 16,000 × *g* for 10 min at 4 °C, after which the supernatant was transferred to a new clean Eppendorf tube. To assay total AsA levels, 20 μl of the supernatant was transferred to the wells of a microtiter plate containing 20 μl of 5 mmol dithiothreitol (DTT). The plate was incubated for 20 min at 37 °C to convert the oxidized ascorbic acid into the reduced form. Ten microliters of N-ethylmaleimide (NEM; 0.5% w/v in water) was added to remove the excess DTT, followed by incubation for 1 min at room temperature (approximately 25 °C). Eighty microliters of the color reagent (see below) was then added to the mixture, followed by incubation for 1 h at 37 °C. The absorbance was subsequently recorded at 550 nm using an Infinite M200 Pro instrument (Tecan; http://www.tecan.com/). The color reagents were prepared as follows: solution A consisted of 31% orthophosphoric acid, 4.6% (w/v) TCA and 0.6% (w/v) iron chloride (FeCl_3_); solution B consisted of 4% 2,2-dipyridyl (w/v in 70% ethanol); and solutions A and B were mixed at a ratio of 2.75:1 before use. To assay reduced AsA, the DTT and NEM were replaced with the same volume of 0.4 M potassium phosphate buffer (pH 7.4), while the rest of the procedure was the same as that for the total AsA assay.

### Subcellular localization and tissue-specific expression

The coding sequence without the terminal codon of *SlNFYA10* was amplified and inserted into the 5’ terminus of GFP in a pCAMBIA-1302 vector under the control of the CaMV35S promoter to generate 35S::SlNFYA10-GFP constructs. Plasmids were extracted and transferred into tobacco protoplasts, and subcellular localization was observed as described previously^48^.

The promoters of *SlGME1* and *SlNFYA10* were amplified and cloned into a pMV2 vector harboring the *GUS* reporter gene, and the resultant vectors were transferred into *A. tumefaciens* C58 for tomato transformation. GUS staining was performed on seedlings, flowers and mature green fruits.

### Oxidative stress experiments

To evaluate the oxidative resistance of *SlNFYA10*-overexpressing and knockdown lines and the wild type, 80 μl of methylviologen (MV, dissolved in water consisting of 0.1% Tween-20) or water consisting of 0.1% Tween-20 (control) was sprayed onto one-month-old seedlings once a day for three consecutive days. The plant phenotypes were observed one week after spraying, and images were collected.

### DAB staining

MV treatments induce cells to produce excessive ROS, resulting in oxidative damage to plants. Leaves from tomato T_2_ transgenic lines and wild-type plants were treated with MV (or water as a control) to investigate the tomato response to oxidative stress. The MV- and water-treated tomato leaves were soaked in 1 mg/ml DAB solution (pH = 3.8) and then incubated in darkness at room temperature for 24 h. The leaves were then transferred to 96% ethanol and placed in a boiling water bath for 10 min to remove the chlorophyll. The ethanol solution was discarded, and the leaves were soaked with 96% ethanol to remove the floating color (the decolorized leaves were stored in 96% ethanol). The reactive oxygen species of the decolorized leaves were evaluated visually according to the brownness of the leaves.

### Quantification of MDA concentration

Malondialdehyde (MDA), the final product of lipid peroxidation of the cell membrane, reflects the plant response to oxidative stress. Leaves and ripe fruits from T_2_ transgenic lines and wild-type plants were utilized to measure the MDA concentration. Samples were collected and ground to a fine powder in liquid nitrogen, followed by the addition of 3 ml of 5% TCA to 0.2 g of ground tissue; the extraction was performed at room temperature for 30 min. After centrifugation, 2 ml of the supernatant was transferred into a 10 ml tube, after which 2 ml of 0.67% TBA solution was added. After blending, the absorption value of the 200 μl mixture was measured at wavelengths of 450 nm, 532 nm and 600 nm. The supernatant in the standard solution was replaced with ddH_2_O. The concentration of MDA was calculated using the formula *C* = 6.45 × (OD_532_ – OD_600_) – 0.56 × OD_450_.

### Determination of POD activity

Using H_2_O_2_ as an oxidant, POD (peroxidase) catalyzes redox reactions, scavenging H_2_O_2_ from cells. POD activity can reflect the antioxidant capacity and stress tolerance of plants. Leaves and ripe fruits from T_2_ transgenic lines and wild-type plants were harvested to measure POD activity. Samples were collected and ground to a fine powder in liquid nitrogen, followed by the addition of 800 μl of precooled phosphate buffer (50 mmol/l, pH 7.8), mixing and centrifugation. The supernatant (20 μl) was subsequently added to a 1.5 ml reaction mixture, which consisted of 50 ml of phosphate buffer (200 mmol/l, pH 6.0), 28 μl of 2-methoxyphenol and 19 μl of hydrogen peroxide (30%). The absorption value of the mixture was measured at a wavelength of 470 nm.

## Results

### A CCAAT-box transcription factor, SlNFY10, is a candidate regulator of ascorbic acid biosynthesis

*GME1* and *GME2* are two homologous genes in tomato. Both contain six exons and five introns, and the first and fourth introns of *GME2* are longer than those of *GME1*. *GME1*-overexpressing lines showed higher AsA levels than did *GME2-*overexpressing lines^16^. Therefore, we used the promoter of *GME1* to perform a yeast one-hybrid assay in an equalized tomato cDNA library comprising various tissues of roots, leaves, flowers, and fruits at different stages to identify the candidate proteins binding to the promoter of *GME1*. The potential transcription factors or binding proteins involved in *GME1* promoter binding and AsA regulation were isolated (Supplementary Table S1).

**Fig. 1:**
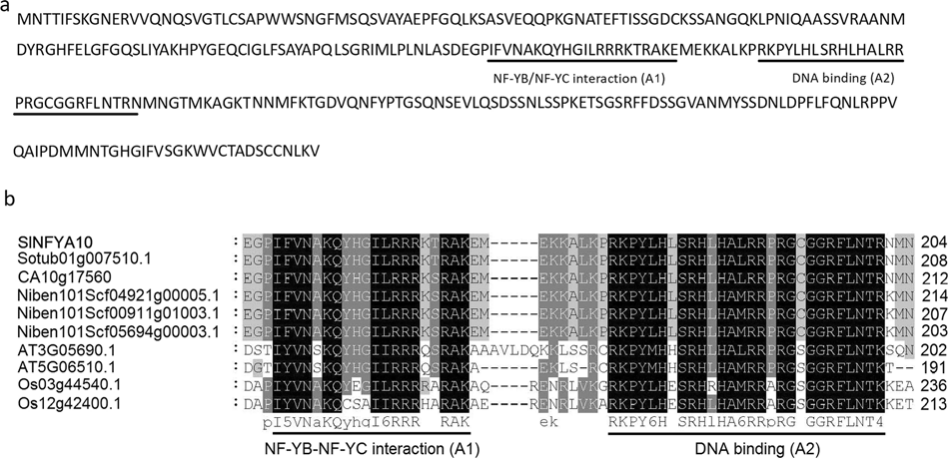
Alignment of the SlNFYA10 sequence with the sequences of its counterparts in plants. **a** Amino acid sequence of SlNFYA10. The NFYB-NFYC interaction (A1) domain and DNA-binding (A2) domain are underlined in black and gray, respectively. **b** Amino acid alignment of the conserved domains of SlNFYA10 and its counterparts from different species. The protein sequences are from tomato (SlNFYA10), potato (*Solanum tuberosum*: Sotub01g007510.1), pepper (*Capsicum annuum*: CA10g17560), tobacco (*Nicotiana attenuata*: Niben101Scf04921g00005.1, Niben101Scf00911g01003.1, and Niben101Scf05694g00003.1), Arabidopsis (AT5G06510.1 and AT3G05690.1), and rice (*Oryza sativ*a L.: OS_03g44540.1 and OS_12g42400.1)

Among the candidate binding proteins, the only annotated transcription factor, a CCAAT-box transcription factor (Solyc01g006930.2) that belong to the NFYA family and is a subunit of the NF-Y complex, was further investigated. The sequence of Solyc01g006930.2 was queried via BLAST against the SOL Genomics Network database (http://solgenomics.net/) and mapped onto chromosome 1. It contains an open reading frame of 936 bp, encoding 312 aa, with an NFYB/NFYC interaction domain (A1: 145–163 aa) and a CCAAT-binding domain (A2: 175–198 aa) (Fig. 1a), and its sequence is highly homologous with that of its counterparts from other plant species (Fig. 1b). According to phylogenetic evolution and annotation, the isolated CCAAT-box transcription factor is hereafter referred to as SlNFYA10.

### SlNFYA10 binds to the *SlGME1* promoter in plants and yeast

**Fig. 2:**
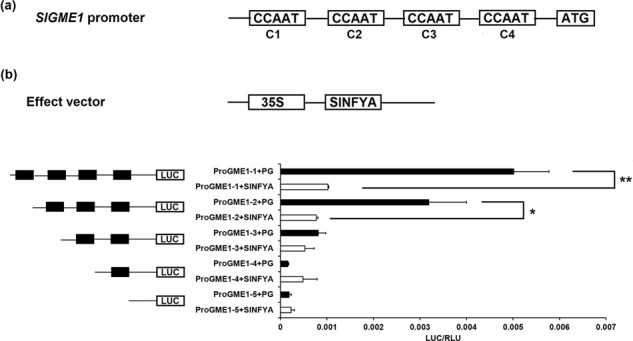
Interaction of SlNFYA10 with the *SlGME1* promoter according to a dual-luciferase assay. **a** CCAAT-box distribution within the *SlGME1* 2-kb promoter. There are four cis-elements in the 2-kb promoter region of *SlGME1*. Four CCAAT elements are represented by black boxes and are named C1, C2, C3, and C4. **b** Dual-luciferase assay between the *SlGME1* promoter and SlNFYA10. The full sequence and serial truncated versions of the *SlGME1* promoter were ligated into pGreenII 0800-LUC as a reporter vector, and *SlNFYA10* was ligated into pGreenII 62-SK as an effector vector. PG, the pGreenII 62-SK empty vector with serial *SlGME1* truncated promoters, was used as a control. LUC luciferase activity. RLC *Renilla* luciferase. The data are means ± SEs (*N* = 3)

To test the hypothesis that SlNFYA10 could directly bind to the CCAAT-box in the promoter of *GME1*, we analyzed the 2-kb promoter fragment of *GME1* through the PLACE (http://www.dna.affrc.go.jp/PLACE/signalscan.html) and PlantCARE (http://bioinfor-matics.psb.ugent.be/webtools/plantcare/html/) databases^49^. We found four potential target elements, two elements of CCAAT and two of ATTGG, that were named C1 and C2 as well as C3 and C4, respectively (Fig. 2a).

NFYA proteins have been previously reported to recognize and bind to CCAAT-box cis-elements, thus regulating the expression of downstream genes. To verify whether SlNFYA10 recognizes and binds to one of these cis-elements, we generated five constructs containing serial truncated promoters (Fig. 2a). These constructs were then transiently expressed in tobacco (*Nicotiana benthamiana*) leaves by *A. tumefaciens* (GV2260)-mediated transformation. Plant agro infiltration showed that the truncation of the promoter successively reduced the promoter capability and luciferase (LUC) activity. When coinfiltrated together with SlNFYA10, the *SlGME1* promoter with four boxes (proGME1–1: C1-C4) and three boxes (proGME1–2: C2-C4) showed significantly reduced LUC activity compared to that of the control. However, the promoter fragments with two boxes (proGME1–3: C3-C4), one box (proGME1–4: C4) or no box (proGME1–5) did not show significant differences in LUC activity compared to that of the control (Fig. 2b). These results indicated that SlNFYA10 mainly binds to C1 and C2 boxes or only C2 boxes.

**Fig. 3:**
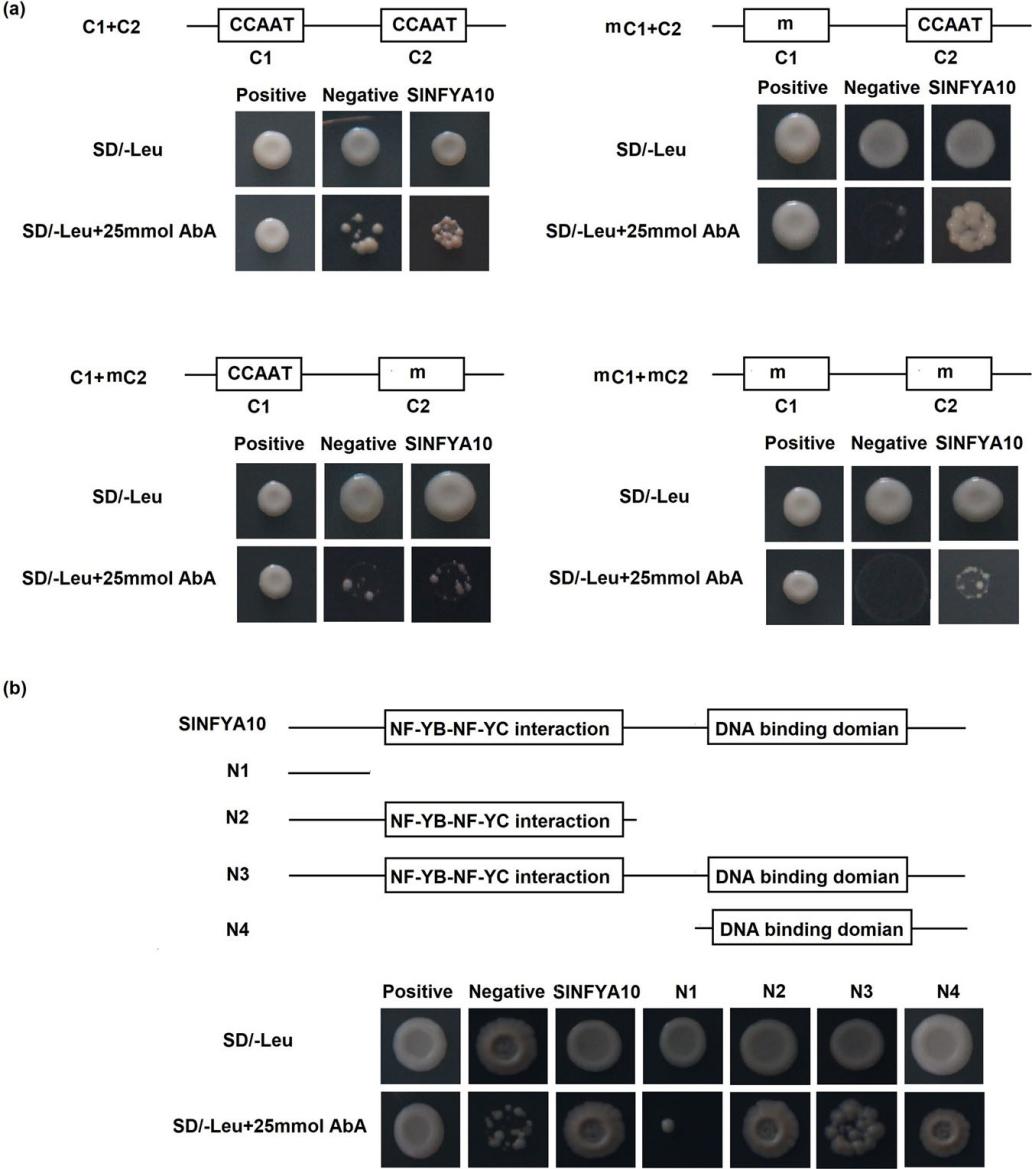
SlNFYA10 binds to the *SlGME1* promoter. **a** The *SlGME1* promoter fragment harboring C1 and C2 elements interacts with SlNFYA10 according to a yeast one-hybrid assay. The C1 and C2 elements along with their mutated version constituted four combinations (the first contained native C1 and C2 elements, the second contained mutated C1 and native C2 elements, the third contained native C1 elements and mutated C2 elements, and the fourth contained mutated C1 and C2 elements). These promoter segments were inserted into the pAbAi vector as bait, and *SlNFYA10* was inserted into the pGADT7 vector as prey. The bait and prey vectors were cotransformed together along with a negative control (bait-pGADT7) or positive control (p53-AbAi/pGAD-p53) into yeast on selective media with or without antibiotics (25 ng/ml AbA). **a** The interaction between C2 elements and serial *SlNFYA10* domains according to a yeast one-hybrid assay. On the basis of its structural characteristics, *SlNFYA10* was divided into four segments and inserted into the pGADT7 vector to serve as prey, and the *SlGME1* promoter contained the whole C2 element as bait. The transformed yeast cells were grown on selective media with or without antibiotics (25 ng/ml AbA)

To identify the core sequence of the *SlGME1* promoter bound by SlNFYA10, constructs with truncated promoters containing C1 and C2 boxes were used in a yeast one-hybrid assay (Fig. 3a). In addition, we artificially mutated the two boxes and transferred them into yeast Y1H Gold strains. The results showed that the *SlGME1* promoter containing native C1 and C2 boxes could be bound by SlNFYA10 and that fragments with mutant C1 boxes and native C2 boxes could be bound by SlNFYA10. However, the promoter fragments with native C1 and mutant C2 boxes or both mutant C1 and C2 boxes could not be bound by SlNFYA10. Taken together, these results indicated that SlNFYA10 may preferably bind to the C2 box of the *SlGME1* promoter.

To further investigate the interaction characteristics between SlNFYA10 and the core elements of the *SlGME1* promoter, we analyzed the protein structure of SlNFYA10 via the NCBI database (https://www.ncbi.nlm.nih.gov/) and found that there are two domains: a DNA-binding domain and an NFYB/NFYC interaction domain. According to the protein structure of SlNFYA10, we divided the coding sequence into four segments (N1–N4) and inserted them into pGADT7 vectors. We used a yeast one-hybrid assay to test the interaction of these different SlNFYA10 segments with the C2 box of the *SlGME1* promoter. The results demonstrated that N2, N3, and N4 could recognize and bind to the C2 box of the *SlGME1* promoter, while the N1 fragment could not (Fig. 3b), indicating that the C-terminus of NFYA is crucial for binding to the *SlGME1* promoter.

**Fig. 4:**
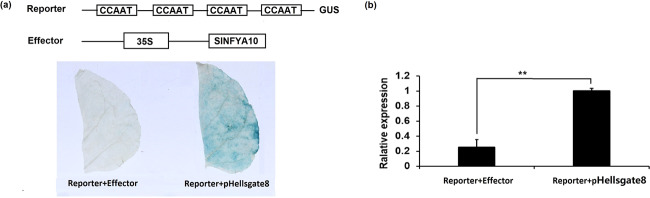
Transcriptional activity of SlNFYA10 in plants. Schematic diagram of the two vectors used for the transient expression assays. The *GUS* gene driven by the *SlGME1* promoter (pro*SlGME*1::GUS), along with the *SlNFYA10*-overexpressing vector as the effector (35S::*SlNFYA10*), were coinfiltrated into tobacco leaves. A *GUS* reporter plus pHELLSGATE8 empty vector was utilized as a control. GUS staining (**a**) and *GUS* gene expression (**b**) were evaluated. The asterisks indicates significant differences (**P ≤ 0.01)

We subsequently inserted the promoter of *SlGME1* into pMV2 to generate pro*SlGME1*::GUS and cloned *SlNFYA10* into pHELLSGATE8 to generate a pro35S::*SlNFYA10* overexpression construct. We transferred the two vectors into *A. tumefaciens* (GV2260) and mixed them together for agro infiltration of tobacco leaves, after which GUS staining and *GUS* gene expression were evaluated. The tobacco leaves infiltrated with pro*SlGME1*::GUS and pHELLSGATE8 exhibited blue GUS activity, while replacement of pHELLSGATE8 with pro35S::*SlNFYA10* led to significantly reduced GUS expression. This was confirmed by qPCR-based detection of *GUS* gene expression in the infiltrated leaves. Taken together, these results indicated that SlNFYA10 can interact with and negatively regulated the *SlGME1* promoter (Fig. 4).

A GAL4/UAS-based assay was also performed, where GDBD binds to six copies of UAS to activate GUS expression^50,45^. The SlNFYA10 coding sequence was fused to GDBD to generate a GDBD-SlNFYA10 fusion protein, which was then coexpressed together with 35S-UAS-GUS in tobacco leaves. Histochemical staining showed that GUS expression was prominently repressed in the leaves cotransformed with GDBD-SlNFYA10 compared with the leaves cotransformed with 35S-GDBD (Supplementary Fig. S1), suggesting that SlNFYA10 might act as a transcriptional repressor.

### SlNFYA10 regulates AsA biosynthesis by modulating *SlGME1* expression

**Fig. 5:**
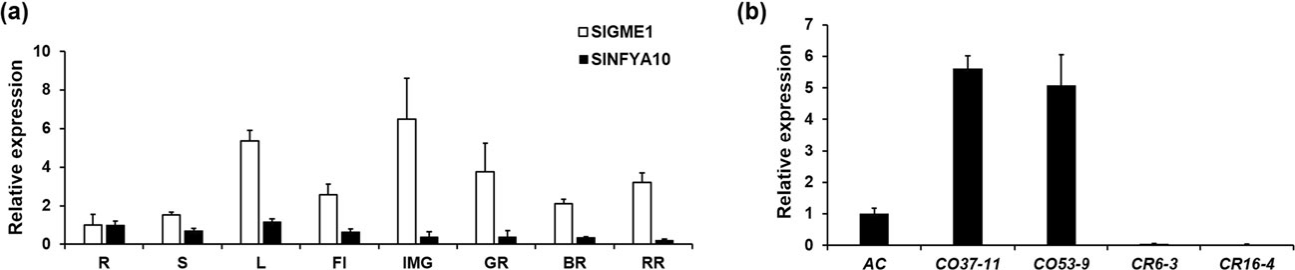
Relative expression of *SlGME1* and *SlNFYA10* in tomato. **a** Tissue expression of *SlGME1* and *SlNFYA10*. *SlGME1* and *SlNFYA10* expression in different organs: R roots, S stems, L leaves, Fl flowers, IMG immature fruits, MG mature green fruits, BR breaker-stage fruits, RR red ripe fruits. **b** Relative expression level of *SlNFYA10* in transgenic lines. qPCR-based analysis was performed on two *SlNFYA10*-overexpressing lines (CO37–11, CO53–9) and two RNAi lines (CR6–3, CR16–4). The data are presented as the means ± SEs

**Fig. 6:**
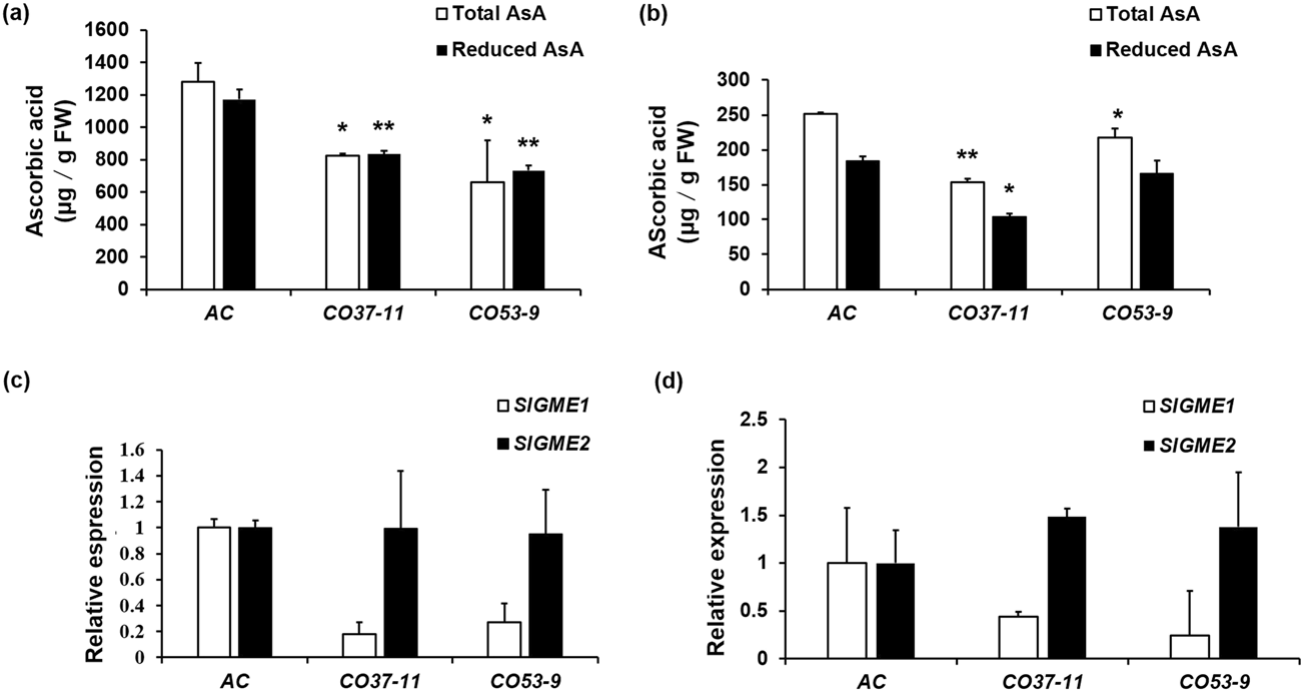
Ascorbic acid levels and *SlGME* expression in *SlNFYA10* transgenic tomato lines. **a** Total and reduced ascorbic acid content in young leaves of *SlNFYA10*-overexpressing lines. **b** Total and reduced ascorbic acid content in red ripe fruits of *SlNFYA10*-overexpressing lines. **c** Relative expression levels of *SlGME1* and *SlGME2* in young leaves of *SlNFYA10*-overexpressing lines. **d** Relative expression levels of *SlGME1* and *SlGME2* in red ripe fruits of *SlNFYA10*-overexpressing lines. The data are the means ± SEs (*N* = 3), and the asterisks indicate significant differences (**P ≤ 0.01; *P ≤ 0.05)

We constructed *SlNFYA10*-overexpressing (CO) and RNAi knockdown (CR) tomato lines. The overexpressing lines exhibited significantly higher *SlNFYA10* expression than did the wild-type control, while the CR lines showed a decrease in expression. The expression of *SlNFYA10* in the leaves of the CO-37–11 and CO-53–9 lines was 5.6- and 5.0-fold that in wild-type leaves, while the CR-6–3 and CR-16–4 lines showed decreased expression (4% and 16% lower than that of the wild-type control, respectively) (Fig. 5b). The AsA levels of the transgenic lines and the wild-type plants were assayed, and the results revealed a significant decrease in the leaves and red ripe fruits of the overexpression lines (Fig. 6a, b), while the RNAi transgenic lines showed no difference in AsA levels (Supplementary Fig. S2a, b). This might be due to the functional redundancy of the NFYA gene family members, for which suppression of a single gene member might be compensated by alternative gene members.

It was previously reported that overexpression of the AsA biosynthetic gene *SlGME1* in tomato caused an increase in AsA levels^16^. We found that enhanced expression of *SlNFYA10* modulated its target gene, *SlGME1*, in *SlNFYA10*-overexpressing plants. The expression of *SlGME1* was significantly decreased in the leaves and red ripe fruits of the overexpression lines compared with the wild type (Fig. 6c, d). However, *SlGME1* expression was not altered in the RNAi lines (Supplementary Fig. S2c, d). In the *SlNFYA10* transgenic lines, *SlGME2* expression was unchanged (Fig. 6c, d and Supplementary Fig. S2c, d). Yeast one-hybrid assays between the *SlGME2* promoter and SlNFYA10 consistently showed no interaction (Supplementary Fig. S3). The negative regulatory effects of SlNFYA10 on *SlGME1* expression were also supported by the fact that SlNFYA10 and *SlGME1* expression abundance exhibited opposite change patterns among the different tissues (Fig. 5a). In summary, SlNFYA10 exerts a specific regulatory effect on *SlGME1* and subsequently negatively modulates AsA accumulation.

### SlNFYA10 regulates multiple sites in the d-mannose/l-galactose pathway

To identify whether SlNFYA10 regulates the expression of other biosynthetic genes in the d-Man/l-Gal pathway, we performed qPCR on the leaves and red ripe fruits of the *SlNFYA10* transgenic lines. In the leaves and red ripe fruits, the expression of *SlGGP1* was decreased in the CO lines and enhanced in the CR lines (Supplementary Fig. S4). The pattern of *SlGGP1* expression modulated by SlNFYA10 in the transgenic lines was similar to that of *SlGME1* expression. These results indicate that SlNFYA10 might regulate AsA biosynthesis at multiple sites.

**Fig. 7:**
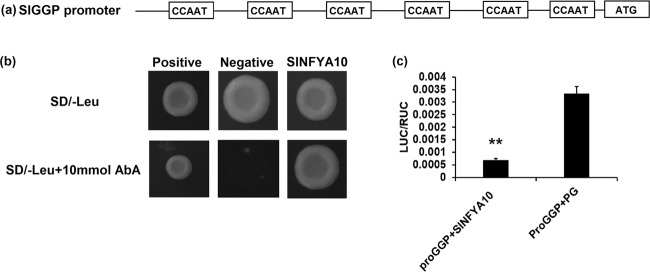
*SlNFYA10* binding to the *SlGGP1* promoter. **a** Analysis of CCAAT elements in the 1.5 kb *SlGGP1* promoter region. **b** Interaction between the *SlGGP1* promoter and *SlNFYA10* in a yeast one-hybrid assay. The *SlGGP1* promoter was inserted into a pAbAi vector as bait, and *SlNFYA10* was inserted into pGADT7 as prey. Cotransformed yeast cells were grown on selective media with or without 10 ng/ml AbA. **c** Transient expression in tobacco leaves. The *SlGGP1* promoter was ligated into pGreenII 0800-LUC as a reporter vector, and *SlNFYA10* was ligated into pGreenII 62-SK as an effector vector. PG, pGreenII 62-SK empty vector

We retrieved the sequences of the promoter fragments of AsA biosynthetic genes from the SOL Genomics Network database and analyzed their cis-elements using the PLACE program. Some genes (e.g., *SlGGP*, *SlGME2*, *SlGLDH*) were predicted to contain CCAAT-boxes that might be bound with NFYA proteins (Supplementary Table S4). We tested the potential interaction between these gene promoters and SlNFYA10 by yeast one-hybrid assays. The yeast one-hybrid results showed that SlNFYA10 could bind to the promoter of *SlGGP1* in yeast (Fig. 7a, b). The *SlGGP1* promoter was then inserted into a pGreenII 0800-LUC vector for transient expression in tobacco. Compared with those infiltrated with pro*SlGGP1* and pGreenII 62-SK, tobacco leaves coinfiltrated with pro*SlGGP1* and SlNFYA10 showed significantly decreased LUC activity (Fig. 7c), suggesting negative regulatory effects of SlNFYA10 on *SlGGP1* as well as *SlGME1*.

### Nuclear factor SlNFYA10 showed tissue-specific expression coincident with *SlGME1*

**Fig. 8:**
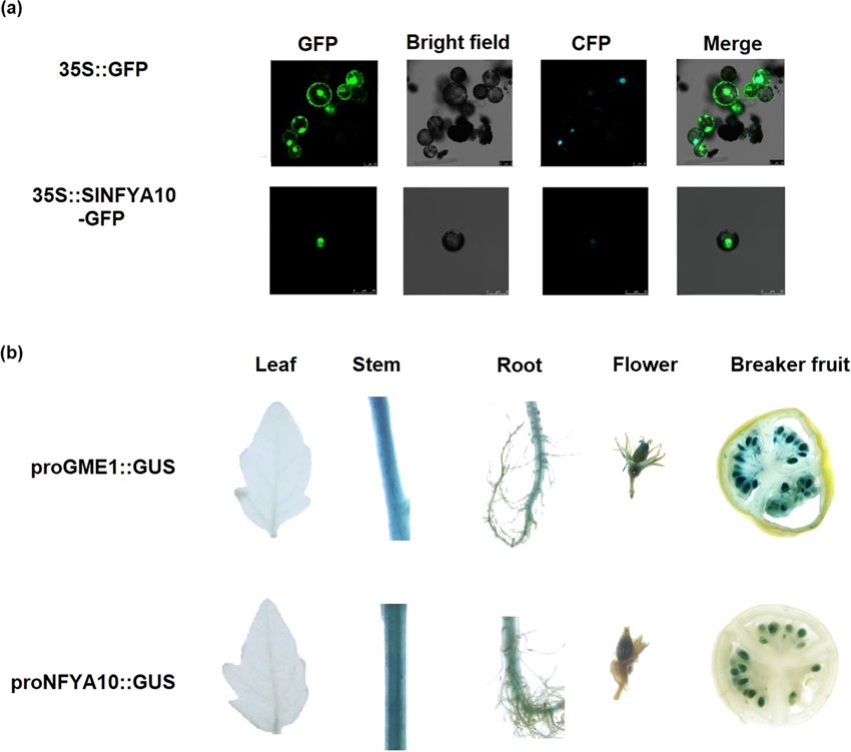
Nuclear localization of SlNFYA10 and its co-occurrence with SlGME1. **a** Subcellular localization of SlNFYA10 in tobacco protoplasts. A *GFP* tag was fused to *SlNFYA10*, and the fusion protein was transiently expressed in tobacco protoplasts followed by fluorescence observations via confocal microscopy. CFP nuclear marker fluorescence. **b** GUS staining of transgenic lines harboring pro*SlGME1*::GUS and pro*SlNFYA10*::GUS. The promoters of *SlGME1* and *SlNFYA10* were inserted into the pMV2 vector driving the *GUS* gene, and GUS staining was performed on the roots, stems, leaves, flowers and breaker-stage fruits

To observe the expression patterns of *SlGME1* and SlNFYA10, 2-kb length promoters of *SlGME1* and *SlNFYA10* were inserted into GUS expression vectors separately. The two GUS constructs were subsequently transformed into the tomato AC cultivar. GUS staining was performed on the roots, stems, leaves, flowers, and breaker fruits of pro*SlGME1*::GUS and pro*SlNFYA10*::GUS transgenic lines. From these staining results, we found that both *SlGME1* and *SlNFYA10* are expressed in the roots, stems, flowers and seeds (Fig. 8b).

To investigate the subcellular localization of *SlNFYA10*, a *SlNFYA10*::GFP fusion protein was constructed and transiently expressed in tobacco protoplasts, followed by observations via confocal microscopy. The results showed that SlNFYA10 was localized in the nucleus (Fig. 8a), which is consistent with the fact that it is a nuclear transcription factor.

### *SlNFYA10* modulates reactive oxygen species accumulation and the stress response

**Fig. 9:**
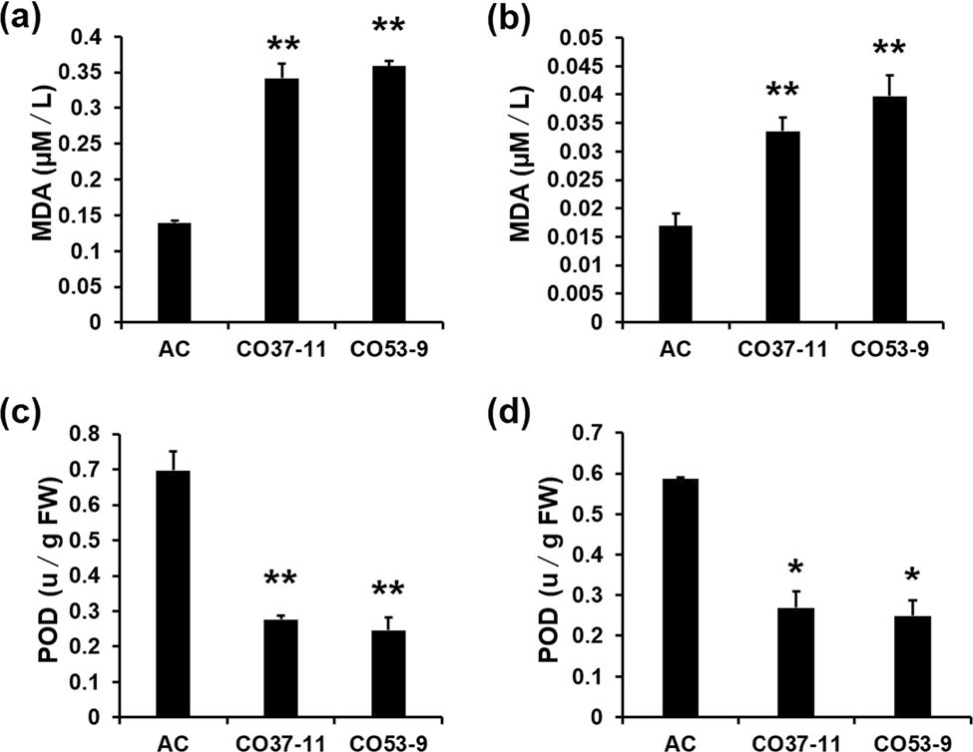
Antioxidant capacity of SlNFYA10 transgenic lines. The MDA concentration was determined in the leaves (**a**) and ripe fruits (**b**) of *SlNFYA10*-overexpressing lines and the wild-type control (AC). POD activity was determined in the leaves (**a**) and red ripe fruits (**b**) of *SlNFYA10*-overexpressing lines and the wild-type control. The data are the means ± SEs (*N* = 3), and the asterisks indicate significant differences (**P ≤ 0.01; *P ≤ 0.05)

AsA is an antioxidant that scavenges reactive oxygen species and protects plants from oxidative stress. We measured the MDA concentration and POD activity in the leaves and fruits of transgenic and wild-type plants. The results showed that the MDA concentration in the overexpressing lines were significantly higher than those in the wild type (Fig. 9a, b), while there was not much difference between the RNAi lines and the control (Supplementary Fig. S5a, b). POD activity was also evaluated in the leaves and fruits, and the results showed that the POD activity was significantly lower in overexpression lines than in the wild-type and RNAi lines (Fig. 9c, d and Supplementary Fig. S5c, d).

To test whether alteration of *SlNFYA10* expression exerts an effect on the plant stress response, transgenic lines and wild-type plants were treated with MV to induce oxidative stress. DAB staining was subsequently carried out to detect ROS in the leaves. We found that the color of the leaves of the *SlNFYA10*-overexpressing lines was darker than that of the RNAi lines and the control, indicating that the leaves of the *SlNFYA10*-overexpressing lines were more severely affected by oxidative stress (Supplementary Fig. S6). Thus, the AsA levels and ROS scavenging capability decreased in *SlNFYA10*-overexpressing lines. It should be noted that suppression of *SlNFYA10* did not significantly alter the ROS accumulation (Supplementary Fig. S6), MDA concentration or POD activity (Supplementary Fig. S5), suggesting functional redundancy among members of the *NFYA* gene family. However, the antioxidant activity of RNAi lines under stress conditions deserves further investigation.

## Discussion

Transcription factors or regulators of AsA biosynthesis have been reported, most of which focus on the regulation of *SlGMP*. Limiting *SlGME1* can exert a large effect on AsA accumulation. Moreover, overexpression and knockdown of *SlGME1* can affect AsA content^17^. In the present study, we used the promoter of *SlGME1* to identify a CCAAT-binding transcription factor, SlNFYA10, which could collectively modulate *SlGME1* and *SlGGP1* expression and alter the AsA content in tomato.

The CCAAT-binding transcription factor also named Nuclear Factor Y (NFY) and Heme activator protein contains three subunits: NFYA, NFYB and NFYC^34,51^. NFYAs bind to the CCAAT-box in promoters and, in conjunction with NFYB and NFYC subunits, form the NFY transcription complex to modulate the expression of downstream genes. *SlNFYA10* belongs to the NFY family, whose members have conserved NFYB/NFYC interaction domains and CCAAT-binding domains. There are also conserved NFYB/NFYC interaction domains and DNA-binding domains in SlNFYA10 (Fig. 1a). NFYs have been demonstrated to regulate plant growth and development and stress responses: *NFYA5* overexpression alleviates moisture loss and drought sensitivity^39^; overexpression of the wheat *NFYA10* gene increases plant sensitivity to salinity, as judged from seed germination and root growth^40^; and NFYs can positively regulate flowering by interacting with the *FT* (FLOWERING LOCUS) promoter^41^.

When the *SlGME1* promoter was used as bait for yeast one-hybrid assays, a CCAAT-box transcription factor (*SlNFYA10*) was isolated. Among the four CCAAT-boxes predicted in the *SlGME1* promoter, only the second CCAAT-box could interact with *SlNFYA10*, as revealed by luciferase and yeast one-hybrid assays (Figs. 2 and 3). GUS staining and expression quantification through transient expression in tobacco (Fig. 4) and the expression patterns of *SlNFYA10* and *SlGME1* (Fig. 5) indicated that there may be a negative regulation effect between *SlNFYA10* and *SlGME1*. Compared with the control plants, the *SlNFYA10*-overexpressing lines presented a lower AsA content in the leaves and fruits (Fig. 6a, b). These results indicated that *SlNFYA10* exerts a repressive effect on *SlGME1* transcription. The expression of alternative enzymatic genes of the AsA biosynthesis pathway was also evaluated in *SlNFYA10* transgenic lines, among which *SlGGP1* expression pattern was altered in a manner similar to how the pattern of *SlGME1* was altered (Supplementary Fig. S4). The LUC assay and yeast one-hybrid assay showed that SlNFYA10 could bind to the *SlGGP1* promoter (Fig. 7). In addition, the subcellular localization results showed that *SlNFYA10* is located in the nucleus, which is characteristic of nuclear transcription factors (Fig. 8a). The tissue distribution of *SlNFYA10* and *SlGME1* showed that they are mainly expressed in the same stem, flower and seed tissues (Fig. 8b). Oxidative stress tests showed that, compared with the control, the SlNFYA10-overexpressing lines presented less ROS scavenging activity (Fig. 9 and Supplementary Fig. S6), presumably due to lower availability of AsA, which is the substrate required for ascorbate peroxidase function. Taken together, our results demonstrate that NFYA negatively regulates AsA biosynthesis by suppressing *GME1* and *GGP1* in tomato fruit. AsA biosynthesis has been widely investigated, and several transcription factors for biosynthetic genes such as *GMP* have been isolated^30^. In the present work, we confirmed that NFYA could simultaneously regulate *GME* and *GGP* expression. NFY transcription factors have been reported to be involved in a wide range of biological processes^39–41^; however, this is the first evidence that NFYA functions in the regulation of AsA metabolism.

It is worth noting that the silencing of *SlNFYA10* by RNAi did not cause significant alterations in terms of AsA content, *SlGME1* and *SlGGP1* expression, ROS accumulation, MDA concentration or POD activity in the transgenic lines (Supplementary Figs. S2, S4, S5, S6). Furthermore, fruit development or ripening was not significantly altered in *SlNFYA10* RNAi transgenic lines compared to the wild type (Supplementary Fig. S7). The unaltered phenotypes of the *SlNFYA10* RNAi transgenic lines might be due to the functional redundancy of the members of the *NFYA* gene family. Unlike the *NFYA*s encoded by one or two genes in animals and yeast, plants tend to have evolved a relatively larger gene family; e.g., the *NFY* gene family has 10 members in Arabidopsis^52^, 8 in rice^53^, 10 in wheat^54^, and 10 in tomato^55^. Gene replication may contribute to large numbers of gene families that allow functional compensation or buffering against individual mutations, subsequently leading to functional redundancy^56^. The F-box protein family also has a large amount of members, with almost 700 predicted genes in Arabidopsis. However, single mutations or combinations of mutations in pollen-specific F-box genes did not lead to gametophytic lethality, suggesting functional redundancy among the F-box protein-coding genes^57^. In plant viruses such as tobacco etch potyvirus, genotypes with genetic redundancy suffered less from the effects of deleterious mutations and showed relatively minor changes in pathogenicity^58^. Accordingly, the expression of alternative members of the *NFYA* gene family, e.g., *SlNFYA1.1*, *SlNFYA3.1*, and *SlNFYA8*, was significantly higher in the *SlNFYA10* RNAi lines than in the wild type (Supplementary Fig. S8). Thus, we hypothesized that the relative stability of plant performance in the *SlNFYA10* RNAi transgenic lines might be in part due to functional redundancy of *NFYA* gene family members in tomato.

## Conclusion

We used the *SlGME1* promoter to isolate a CCAAT-box transcription factor, SlNFYA10, which could modulate AsA biosynthesis at multiple sites. *SlNFYA10* exerted a negative effect on *SlGME1* and *SlGGP1* expression and AsA accumulation. *SlNFYA10-*overexpressing lines showed a lower antioxidant capacity, with lower AsA content. The identification of potential interacting proteins involved in the heterotrimer complex will provide deeper insight into *SlNFYA10* regulation of AsA biosynthesis.

## Supplementary information

Table S1

Table S2

Table S3

Table S4

Figure S1

Figure S2

Figure S3

Figure S4

Figure S5

Figure S6

Figure S7

Figure S8
