# Supplementary material for: A CCAAT-binding factor, SlNFYA10, negatively regulates ascorbate accumulation by modulating the d-mannose/l-galactose pathway in tomato
Source: Hortic Res. 2020 Dec 1;7:200. doi: 10.1038/s41438-020-00418-6 (PMC7705693; doi:10.1038/s41438-020-00418-6)
Supplement: Supplementary file 1 — Table S1 [file 41438_2020_418_MOESM1_ESM.docx]

| Gene ID Annotated Function |
| --- |
| Solyc01g006930.2 CCAAT-binding transcription factor  Solyc09g090140.2 Malate dehydrogenase  Solyc04g017690.2 Early response to dehydration 15-like protein Solyc03g005760.1 ChlorophyⅡ a/b binding protein  Solyc03g096670.2 Protein phosphatase 2C  Solyc06g065980.2 Nucleic acid binding  Solyc03g098790.1 Kunitz-type protease inhibitor  Solyc01g079880.2 Asparagine synthetase  Solyc04g007890.2 High mobility group protein  Solyc03g097900.2 Ribosomal proteins  Solyc02g086820.2 Carbonic anhydrase |

**Supplemental Table S1 Screening of candidate binding proteins in yeast one-hybrid by *SlGME1* promoter**
