# Supplementary material for: A CCAAT-binding factor, SlNFYA10, negatively regulates ascorbate accumulation by modulating the d-mannose/l-galactose pathway in tomato
Source: Hortic Res. 2020 Dec 1;7:200. doi: 10.1038/s41438-020-00418-6 (PMC7705693; doi:10.1038/s41438-020-00418-6)
Supplement: Supplementary file 2 — Table S2 [file 41438_2020_418_MOESM2_ESM.docx]

**Supplemental Table S2 The primers for vector construction**

**Supplemental Table S2.1 The primers for construction of transformation vectors.**

| Primer | Sequence |
| --- | --- |
| SlNFYA10-OE(CO)-Fw | 5’-CTCGAG GGTGTGAGTTGCTCTTGTATAACCC-3’ |
| SlNFYA10-OE(CO)-Rv | 5’-TCTAGA GCACGGATGAGGTGAGGCT-3’ |
| SlNFYA10-RNAi(CR)-Fw | 5’-CAGGCT TTCATCAGGGGTCGCTAACA-3’ |
| SlNFYA10-RNAi(CR)-Rv | 5’-CTGGGT TAGGCACCGCGTTAATGCTA-3’ |
| proGME1-GUS-Fw | 5’-GAGCTCGCAAACTATAACCAAGAAGACTATT-3’ |
| proGME1-GUS- | 5’-CTCGAG TGAAAAAGCTACACTCAGTCAAA -3’ |
| proNFYA-GUS-Fw | 5’-GAGCTC TGGAATAAAATACAATGGTGATCC-3’ |
| pronFYA-GUS-Rv | 5’-CTCGAGGGTTATACAAGAGCAACTCACACCT-3’ |

**Table S2.2 The primers for transient expression vector construction**

| Primer | Sequence |
| --- | --- |
| SlNFYA10-62sk-Fw | 5’-GGATCC ATGAATACTACTATATTTTCCAAAGG-3’ |
| SlNFYA10-62sk-Rv | 5’-CTCGAG TCATACTTTGAGGTTGCAACAGCTA -3’ |
| proGME1-1-Fw | 5’-GGTACC CAAATACTAGGTGGGCTAGGATGTG-3’ |
| proGME1-2-Fw | 5’-GGTACC GATGGGAGAGGAGCCAATCAG-3’ |
| proGME1-3-Fw | 5’-GGTACC CTCAAGTGTAAACAAGATTTGCAC-3’ |
| proGME1-4-Fw | 5’-GGTACC GTTGTGGAGGTGAGGAGTGAAATC-3’ |
| proGME1-5-Fw | 5’-GGTACC GATGTATGAGTTAAAGCTATGTGAG-3’ |
| proGME1-Rv | 5’-CCATGG TCTGAAAAAGCTACACTCAGTCAAATG-3’ |
| SlNFYA10-PYF503-Fw | 5’-GAATTC ATGAATACTACTATATTTTCCAAAGG-3’ |
| SlNFYA10-PYF503-Rv | 5’-GTCGAC TCATACTTTGAGGTTGCAACAGCTA-3’ |

**Table S2.3 The primers for yeast one hybrid assay**

| Primer | Sequence |
| --- | --- |
| proGME1-Fw | 5’-GGTACC GCAAACTATAACCAAGAAGACTATT-3’ |
| proGME1-Rv | 5’-CTCGAG TCTGAAAAAGCTACACTCAGTCAAA-3’ |
| SlNFYA10-AD-Fw | 5’-GAATTC ATGAATACTACTATATTTTCCAAAGG -3’ |
| SlNFYA10-AD-Rv | 5’-GATCCT TCATACTTTGAGGTTGCAACAGCTA-3’ |
| SlNFYA10-N1-Fw | 5’-GAATTC ATGAATACTACTATATTTTCCAAAGG-3’ |
| SlNFYA10-N1-Rv | 5’-GATCCT CCAAATTCAATGGTAGCATAA-3’ |
| SlNFYA10-N2-Rv | 5’-GATCCT CTTTTTTCTCCATTTCCTTAGC-3’ |
| SlNFYA10-N3-Rv | 5’-GATCCT TCATATTCCTTGTGTTCAAGAAA-3’ |
| SlNFYA10-N4-Fw | 5’-GAATTC ATGGAACTATGAAGGCTGGAA-3’ |
| SlNFYA10-N4-Rv | 5’-GATCCT TCATACTTTGAGGTTGCAACAG-3’ |
| proGGP-Fw | 5’-GGTACC ATTTGTATTGGAAGATGTGTAAATGT-3’ |
| proGGP-Rv | 5’-CTCGAG TTCTTTTTGTGTGTTTTCTTCG-3’ |

**Table S2.4 The primers for subcellular localization**

| Primer | Sequence |
| --- | --- |
| SlNFYA10-GFP-Fw | 5’-ATGGTACCCATGAATACTACTATATTTTCCAAAG-3’ |
| SlNFYA11-GFP-Rv | 5’-ACTAGT TACTTTGAGGTTGCAACAGCTATCT-3’ |
| proGGP-0800-Fw | 5’-GGTACC TTTGTATTGGAAGATGTGTAAATGT-3’ |
| proGGP-0800-Rv | 5’-CCATGG TTACTTCTTTTTGTGTGTTTTCTTC-3’ |
