# Supplementary material for: A CCAAT-binding factor, SlNFYA10, negatively regulates ascorbate accumulation by modulating the d-mannose/l-galactose pathway in tomato
Source: Hortic Res. 2020 Dec 1;7:200. doi: 10.1038/s41438-020-00418-6 (PMC7705693; doi:10.1038/s41438-020-00418-6)
Supplement: Supplementary file 3 — Table S3 [file 41438_2020_418_MOESM3_ESM.docx]

**Supplemental Table S3 The qPCR primers for AsA biosynthetic genes.**

| Gene name * | Gene ID | Forward primer | Reverse primer |
| --- | --- | --- | --- |
| *GPI* | Solyc04g076090 | 5’-TGCTCTTCAAAAGCGTGTCC-3’ | 5’-CGGCAATAAGTGCTCTGTCA-3’ |
| *PMI* | Solyc02g086090 | 5’-TACATTGTGGTGGAACGAGGA-3’ | 5’-ACCCCATTTGGCAAGAACAG-3’ |
| *PMM* | Solyc05g048760 | 5’-TTTACCCTCCATTACATTGCTGA-3’ | 5’-CTTCTTGACTACAGTTTCTCCCA-3’ |
| *GME1* | Solyc01g097340 | 5’-AATCCGACTTCCGTGAGCC-3’ | 5’-CTGAGTTGCGACCACGGAC-3’ |
| *GME2* | Solyc04g077020 | 5’-CCATCACATTCCAGGACCAGA-3’ | 5’-CGTAATCCTCAACCCATCCTT-3’ |
| *GGP1* | Solyc06g073320 | 5’-GAAATCTGGTCTGTTCCTCTGTGA-3’ | 5’-TTCACACACCAACTCCACATTACA-3’ |
| *GMP* | Solyc03g096730 | 5’-AAACCTGAAATCGTGATGTGAGA-3’ | 5’-TGAAGAAGAGGAGAACTGGAAAC-3’ |
| *GP1* | Solyc04g014800 | 5’-AGCCGCTACAAACCCTCATCT-3’ | 5’-TGTCCGCTTTCCATCTCCTAT-3’ |
| *GP2* | Solyc11g012410 | 5’-GGTTAGGTCCCTTCGTATGTG-3’ | 5’-TTTCACAATCACAGCACCACC-3’ |
| *GalDH* | Solyc01g106450 | 5’-CTTCTTACTGAGGCTGGTGGTC-3’ | 5’-AACCTCTTTAACAGACTTCATCCC-3’ |
| *GLDH*  Solyc10g079470 5’-ATTGAGGTTCCCAAGGACATAG-3’ 5’-ACTACTCTTCCTTCTGCTGCTTTA-3’  *MIOX* Solyc12g008650 5’-TGTTATTAGATAGGATGCGGTTT-3’ 5’-AATGTTGAGCCACTTCATGTTCT-3’ | | | |
| *DHAR1* | Solyc05g054760 | 5’-CCTACCTTCGTCTCATTTCCG-3’ | 5’-TGAACAAACATTCTGCCCATT-3’ |
| *Actin* | Solyc11g005330 | 5’-GTCCTCTTCCAGCCATCCA -3’ | 5’-ACCACTGAGCACAATGTTACC-3’ |

* The *DHAR1* for AsA recycling is included and the *Actin* gene was utilized as internal control.
