# Supplementary material for: A CCAAT-binding factor, SlNFYA10, negatively regulates ascorbate accumulation by modulating the d-mannose/l-galactose pathway in tomato
Source: Hortic Res. 2020 Dec 1;7:200. doi: 10.1038/s41438-020-00418-6 (PMC7705693; doi:10.1038/s41438-020-00418-6)
Supplement: Supplementary file 4 — Table S4 [file 41438_2020_418_MOESM4_ESM.docx]

**Supplemental Table S4 Analysis of promoters of biosynthetic genes other than *SlGME1* in D-mannose/L-galactose pathway.** The number of CCAAT/ATTGG elements on 5-kb promoter upstream of AsA biosynthetic genes were analyzed.

| Gene | Numbers of CCAAT/(ATTGG) elements |
| --- | --- |
| *SlPMI*  *SlGPI*  *SlPMM*  *SlGMP1*  *SlGMP2*  *SlGME2*  *SlGGP*  *SlGP*  *SlGalDH*  *SlGLDH*  *SlMIOX*  *SlMDHAR*  *SlDHAR*  *SlcAPX* | 12  10  11  10  15  11  13  10  9  12  8  10  10  10 |
