# Supplementary figures and images for: A CCAAT-binding factor, SlNFYA10, negatively regulates ascorbate accumulation by modulating the d-mannose/l-galactose pathway in tomato
Source: Hortic Res. 2020 Dec 1;7:200. doi: 10.1038/s41438-020-00418-6 (PMC7705693; doi:10.1038/s41438-020-00418-6)

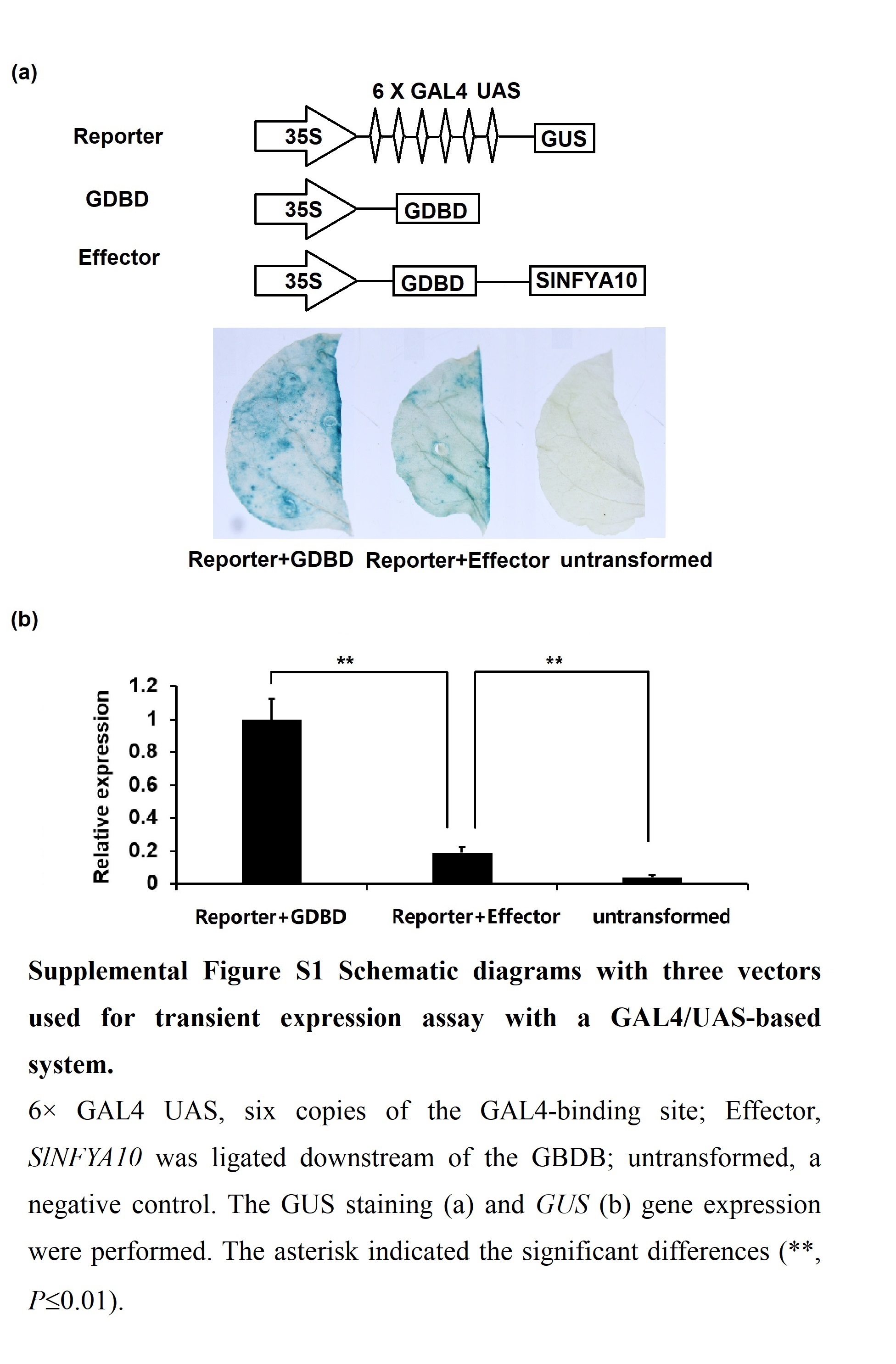

Supplement: Supplementary file 5 — Figure S1 [file 41438_2020_418_MOESM5_ESM.jpg]

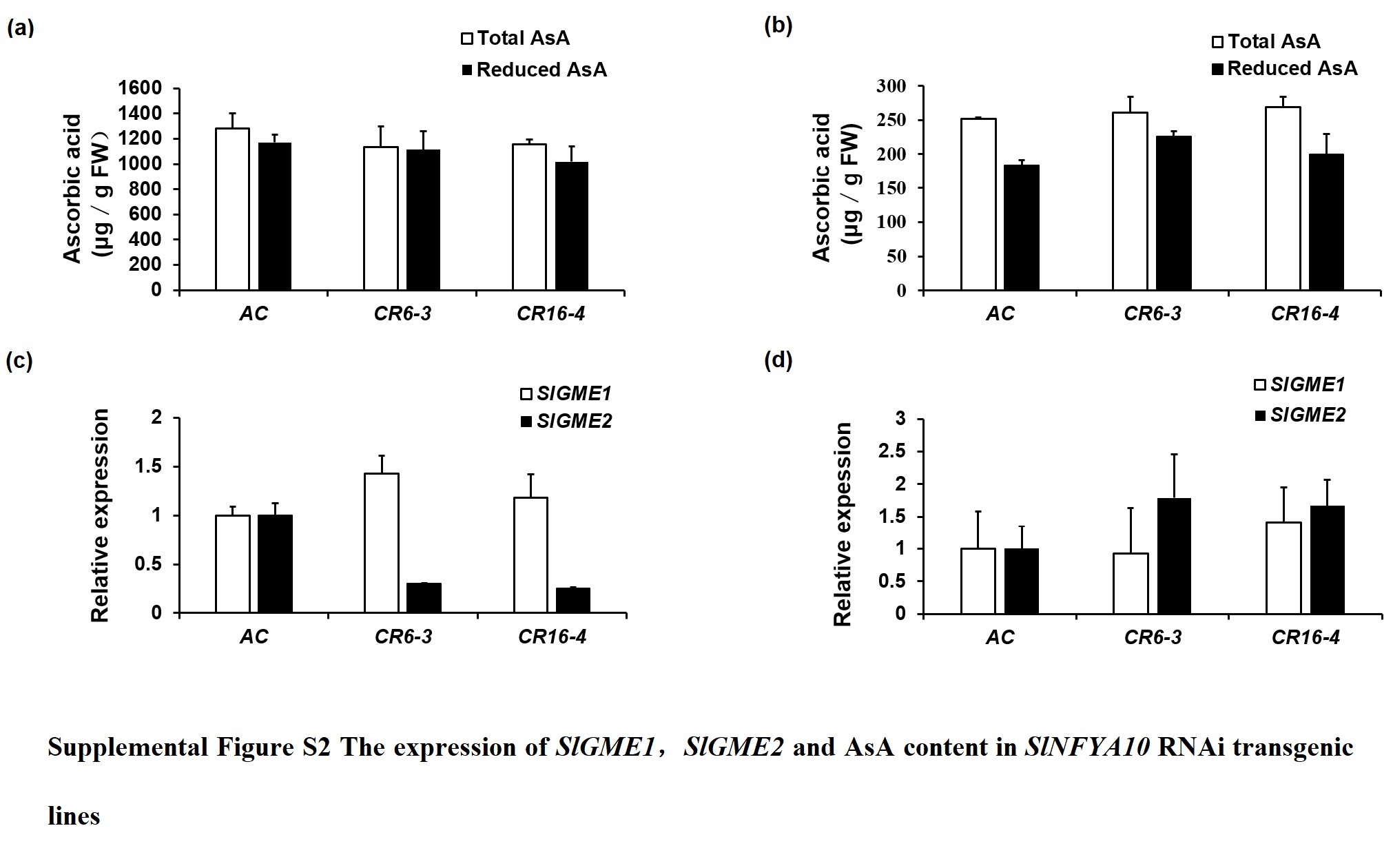

Supplement: Supplementary file 6 — Figure S2 [file 41438_2020_418_MOESM6_ESM.jpg]

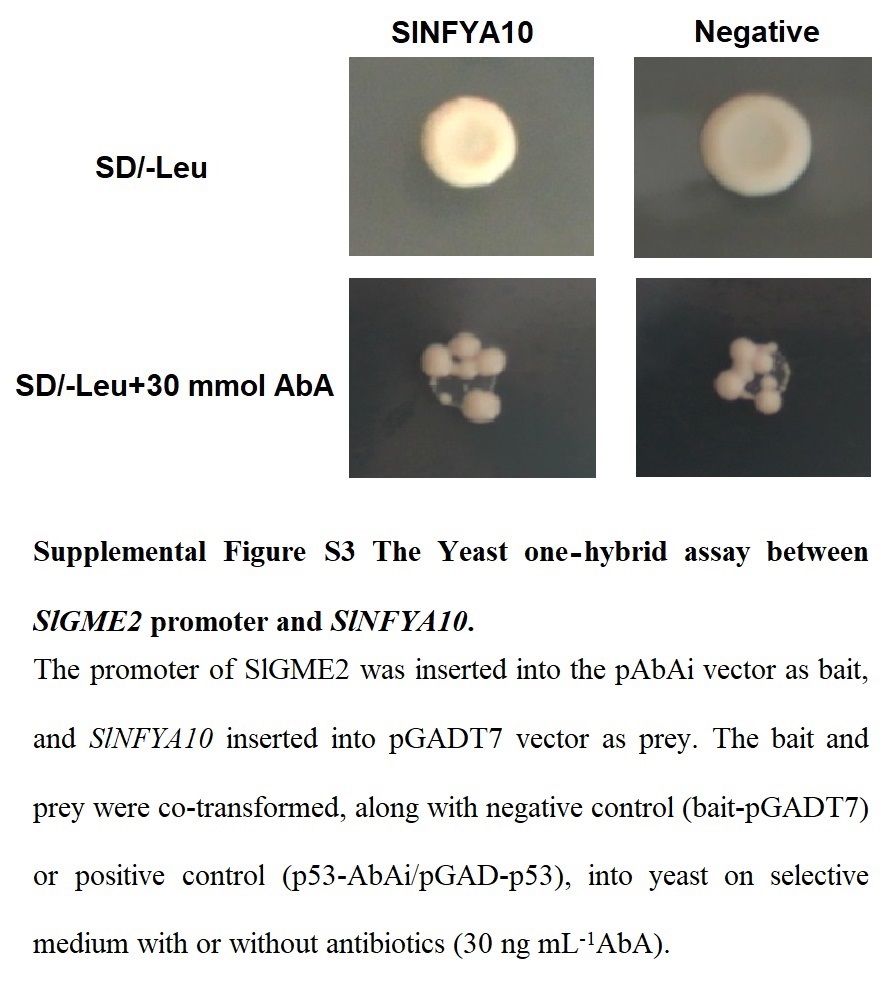

Supplement: Supplementary file 7 — Figure S3 [file 41438_2020_418_MOESM7_ESM.jpg]

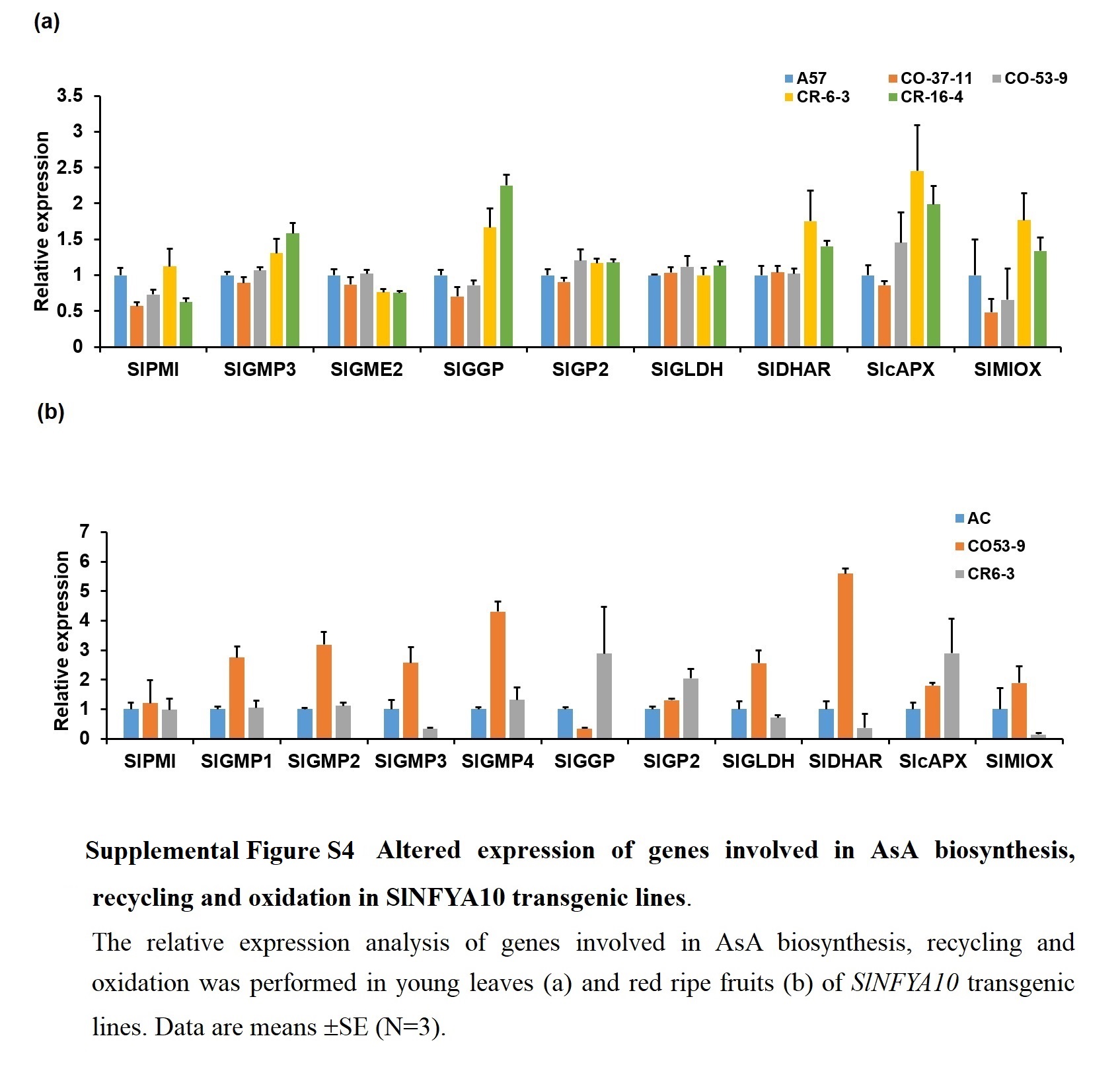

Supplement: Supplementary file 8 — Figure S4 [file 41438_2020_418_MOESM8_ESM.jpg]

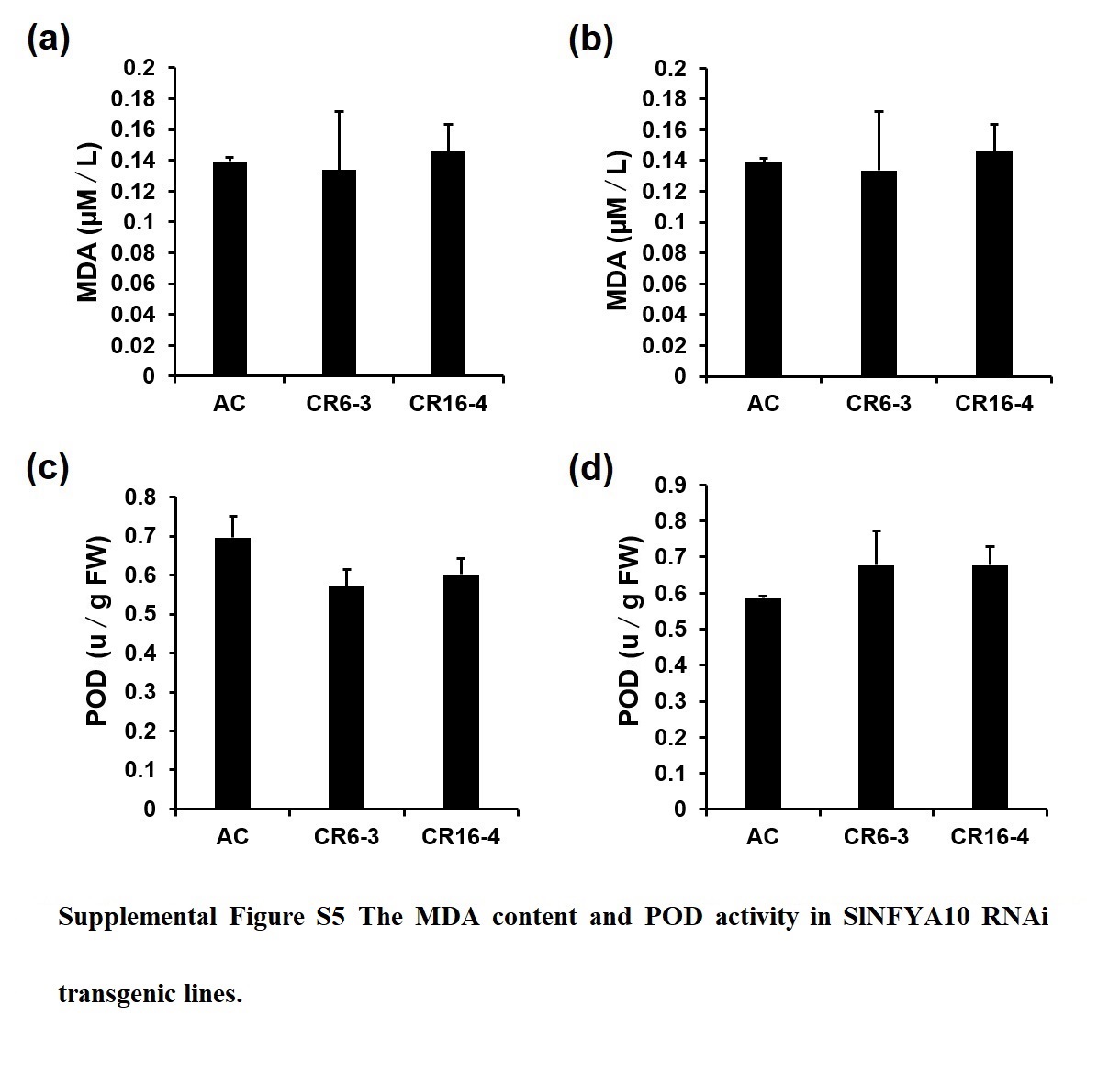

Supplement: Supplementary file 9 — Figure S5 [file 41438_2020_418_MOESM9_ESM.jpg]

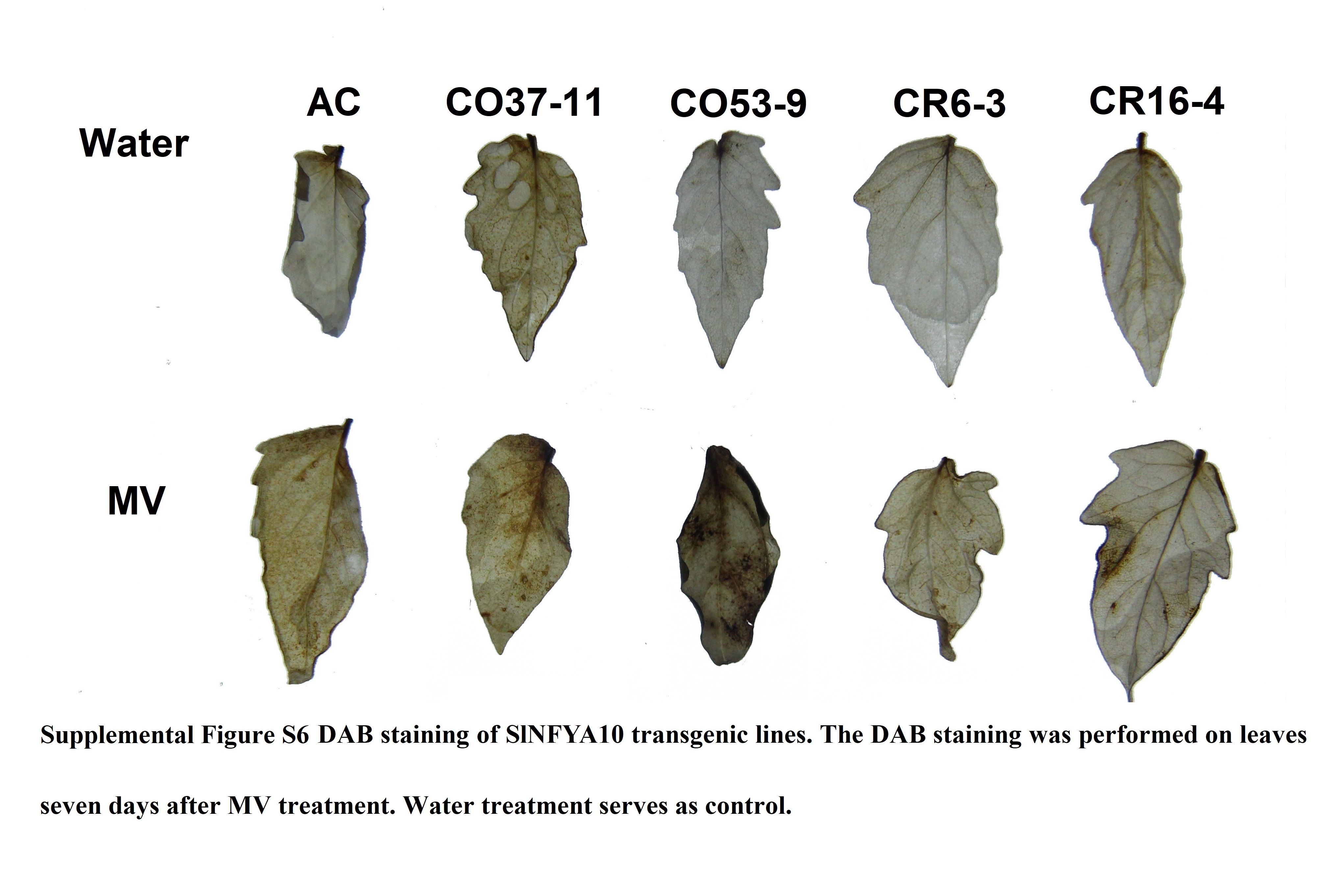

Supplement: Supplementary file 10 — Figure S6 [file 41438_2020_418_MOESM10_ESM.jpg]

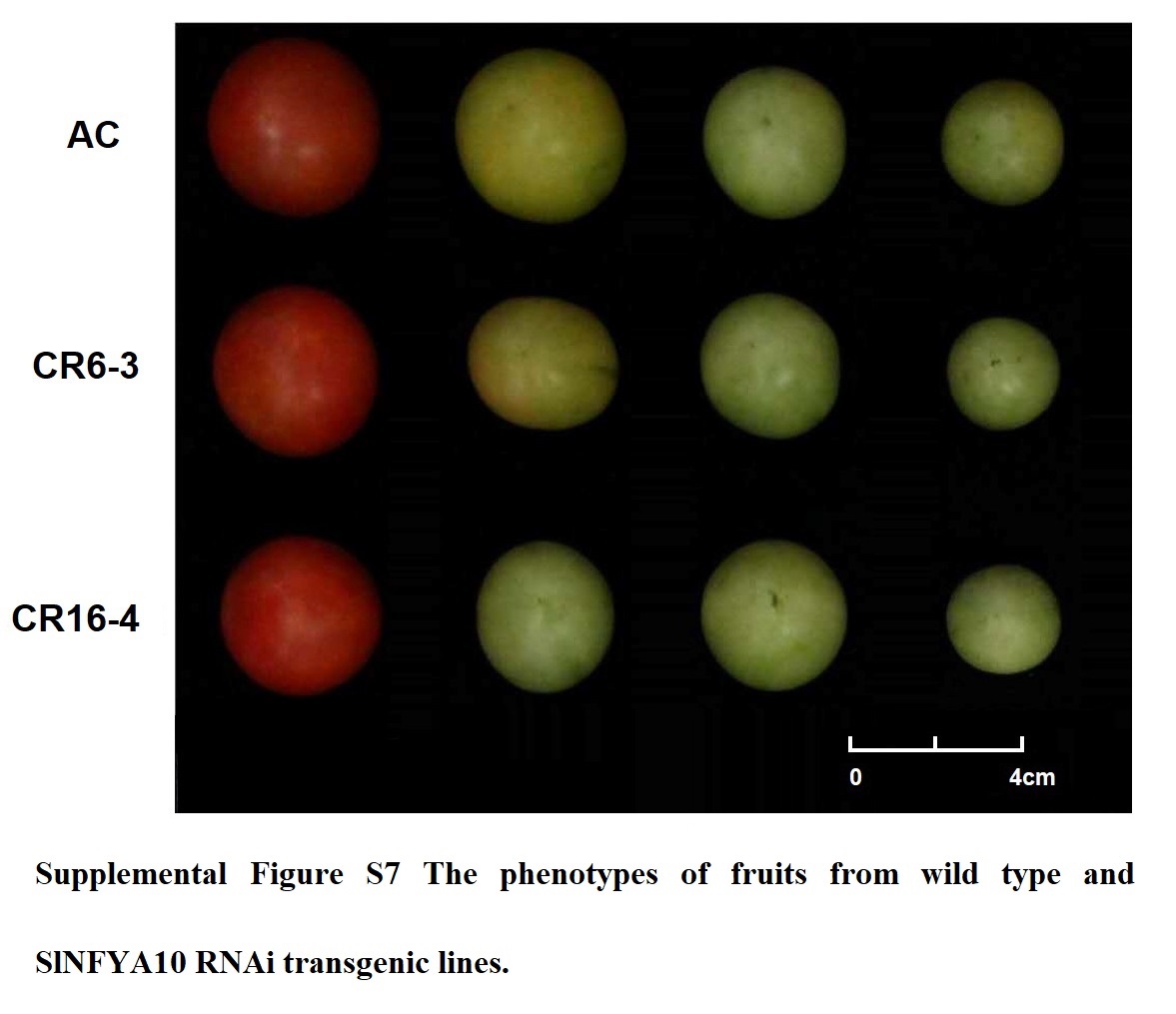

Supplement: Supplementary file 11 — Figure S7 [file 41438_2020_418_MOESM11_ESM.jpg]

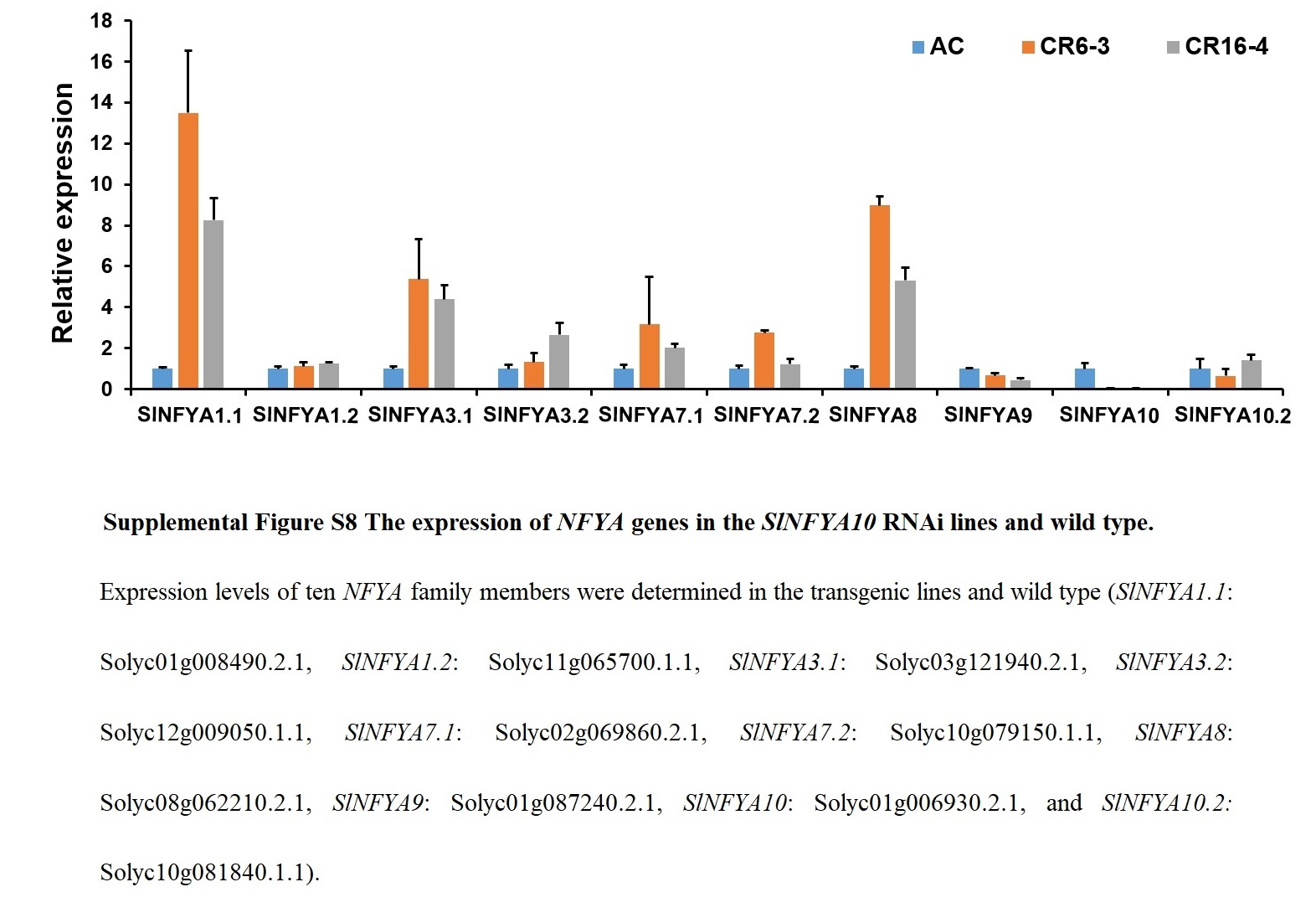

Supplement: Supplementary file 12 — Figure S8 [file 41438_2020_418_MOESM12_ESM.jpg]
